# Detection of Siderophores as a Superior Noninvasive Diagnostic Tool in Unraveling Mixed Fungal Infections

**DOI:** 10.1021/acsomega.5c01914

**Published:** 2025-05-21

**Authors:** Radim Dobiáš, Milan Navrátil, Rutuja H. Patil, Dominika Luptáková, David A. Stevens, Vladimír Havlíček

**Affiliations:** † Department of Bacteriology and Mycology, National Reference Laboratory for Mycological Diagnostics, Public Health Institute in Ostrava, Ostrava 702 00, Czechia; ‡ Institute of Laboratory Medicine, Faculty of Medicine, University of Ostrava, Ostrava 703 00, Czechia; § Department of Haemato-Oncology, University Hospital Ostrava, Czech Republic, Ostrava 703 00, Czechia; ∥ Department of Haemato-Oncology, Faculty of Medicine, University of Ostrava, Ostrava 703 00, Czechia; ⊥ Institute of Microbiology of the Czech Academy of Sciences, Prague 142 00, Czechia; # Division of Infectious Diseases and Geographic Medicine, Stanford University School of Medicine, Stanford, California 94305, United States

## Abstract

Advances in the early diagnosis of systemic mycoses are urgently needed, given the morbidity and mortality of such infections and the correlation between delays in treatment and poor outcomes. We demonstrated the prospective application of liquid chromatography–mass spectrometry in the diagnosis of a mixed fungal infection. In this study, we compared the performance of chest radiography, galactomannan (sGM), and beta-d-glucan (sBDG) serology with a novel diagnostic method based on creatinine-indexed microbial siderophores in urine. A woman with angioblastic T-cell lymphoma presented with neutropenia following allogeneic transplantation. sGM and sBDG remained positive throughout the 28-day intensive care unit stay. A. fumigatus DNA was detected in the induced sputum samples on sampling days 0 and 18. On day 18, a CT scan showed a typical nest sign, and R. microsporus DNA was detected in sputum. The patient was discharged from the hospital on day 28 and expired 7 days later. With our novel strategy based on mass spectrometry, A. fumigatus was consistently detected in the urine from day 0 to the end of the stay by the detection of triacetylfusarinine C (uTafC), an A. fumigatus-specific hydroxamate siderophore. An additional invasive R. microsporus infection was revealed by the detection of a mucoromycete-specific carboxylate siderophore in urine, rhizoferrin (uRhf), from day seven onward. Both creatinine-normalized siderophore indices (uTafC/Cr, uRhf/Cr) were sensitive to antifungal therapy and correlated with fast relapses of the invasive disease in time. This study illustrates how such an early and specific new approach can unravel the complexities of dual fungal infections.

## Graphical abstract

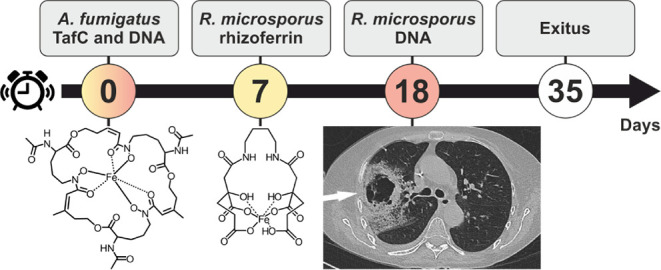

## 1 Introduction

Aspergillus fumigatus (Af) and Mucorales are critical human pathogens designated on the WHO fungal priority list.^1^ Distinguishing between *Aspergillus* and Mucorales infections in clinical settings can be challenging because of the considerable overlap in the at-risk patient populations and the clinical presentation between these infections.^2^ Sensitive and early diagnosis of mixed infections is a significant hurdle.^3^ Mixed mold infections are common and may account for 12% of all invasive fungal episodes in some clinical settings.^4^ As shown by serum qPCR, one-third of all mucoromycoses recorded in a prospective study were mixed *Aspergillus*–Mucorales infections.^4^ Mucorales are resistant to the usual first-line treatments for invasive aspergillosis, and delays in initiating appropriate antifungal therapy for both these two infections are associated with poor clinical outcomes.^5^

Despite some advances in modern fungal diagnostics, the *Aspergillus*–Mucorales coinfections represent a neglected area of polymicrobial infections, where specific and sensitive pathogen analysis is lacking. Molecular tools, including fungal cell-free analytes,^6^ are of great help, but none can reliably distinguish viable from nonviable pathogens based on circulating DNA profiles or cellular antigens.^4^ In contrast, *Af* and Rhizopus microsporus (*Rm*) secrete genus-specific siderophores during active growth.^7^ These molecules reflect not only the viability of the pathogen, but due to their molecular weight and structure, they may easily cross human tissue-blood barriers (Figure 1A), thus facilitating diagnosis, in some cases even noninvasively. As a result, in a mixed non-neutropenic and neutropenic cohort of 13 patients with invasive pulmonary aspergillosis, the triacetylfusarinine C (TafC)/creatinine index from urine samples showed a sensitivity over 92%, in contrast to the 46% sensitivity of the serum galactomannan (sGM) assay.^8^ TafC production in *Af* cells is rapid, detectable within 8 h of infection, with a gradual decline beginning at 48 h, as documented by *in vitro* experiments.^8^ Using a 3 h doubling time and Avogadro’s constant, a single germinating conidium can secrete 10 million molecules of TafC 9 h after inoculation.^9^ This number increases to 100 million molecules after 24 h,^8^ and siderophore excretion in urine is almost immediate.^10^ Biosynthetic output contributes to siderophore concentrations in human body fluids that are detectable in the dynamic range of mass spectrometry.^11^ Similarly, rhizoferrin (Rhf) has been proposed as a specific marker of *Rm* infection.^12^

**Figure 1:**
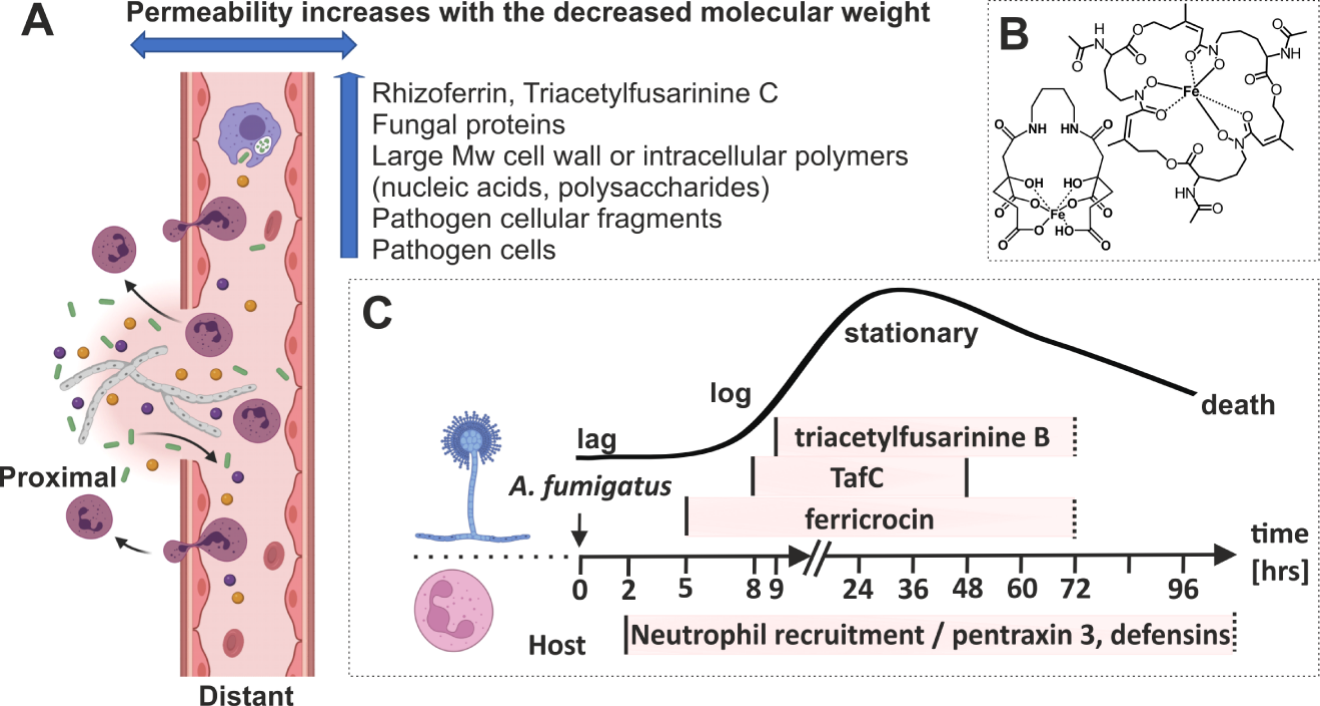
Simulation of tissue-blood barrier permeability and invasive fungal infection development over time. The permeability increases with the decreasing molecular weight of the fungal body constituents (A). Note the proximal (e.g., lung) and distant (e.g., urine) body fluid sampling sites. Chemical structures (B) of triacetylfusarinine C (TafC, top) and Rhf (bottom) fungal siderophores used in this study. The onset (log phase) of aspergillosis (C) can be modeled using *in vitro* data that include triacetylfusarinine B, TafC, and ferricrocin siderophores.^8^ Note the prompt pentraxin 3 secretion correlates with neutrophil recruitment.^14,15^ Dashed lines in biomarker windows (in pink) represent unknown time window closure. The image was in part created in BioRender.com.

In this communication, we show that mixed invasive pulmonary mycosis caused by *Rm* or *Af* can be diagnosed by noninvasive monitoring of Rhf and TafC (Figure 1B) in urine, respectively. At the same time, antifungal drugs were monitored in the urine (Figure 2). The innovative siderophore diagnostic panel was complemented by the assay of pentraxin 3 (Ptx3), an acute-phase protein host factor that differentiates between pulmonary fungal and bacterial diseases.^13^ Ptx3 specifically recognizes galactosaminogalactan, a fungal cell wall polymer,^14^ and is extensively secreted by recruited immune cells that can reach the site of inflammation as early as 2 h after infection.^15^ Modern and novel diagnostic approaches based on the detection of specific fungal and host markers can be of great benefit to physicians, especially intensivists, infectious disease specialists, and pulmonologists, for the life-saving diagnosis of fungal infections. The delineation of *in vitro* kinetic data, namely the timing of microbial log phase and host cell response,^8,15^ is critical for understanding the molecular events occurring at the site of inflammation (Figure 1C).

**Figure 2:**
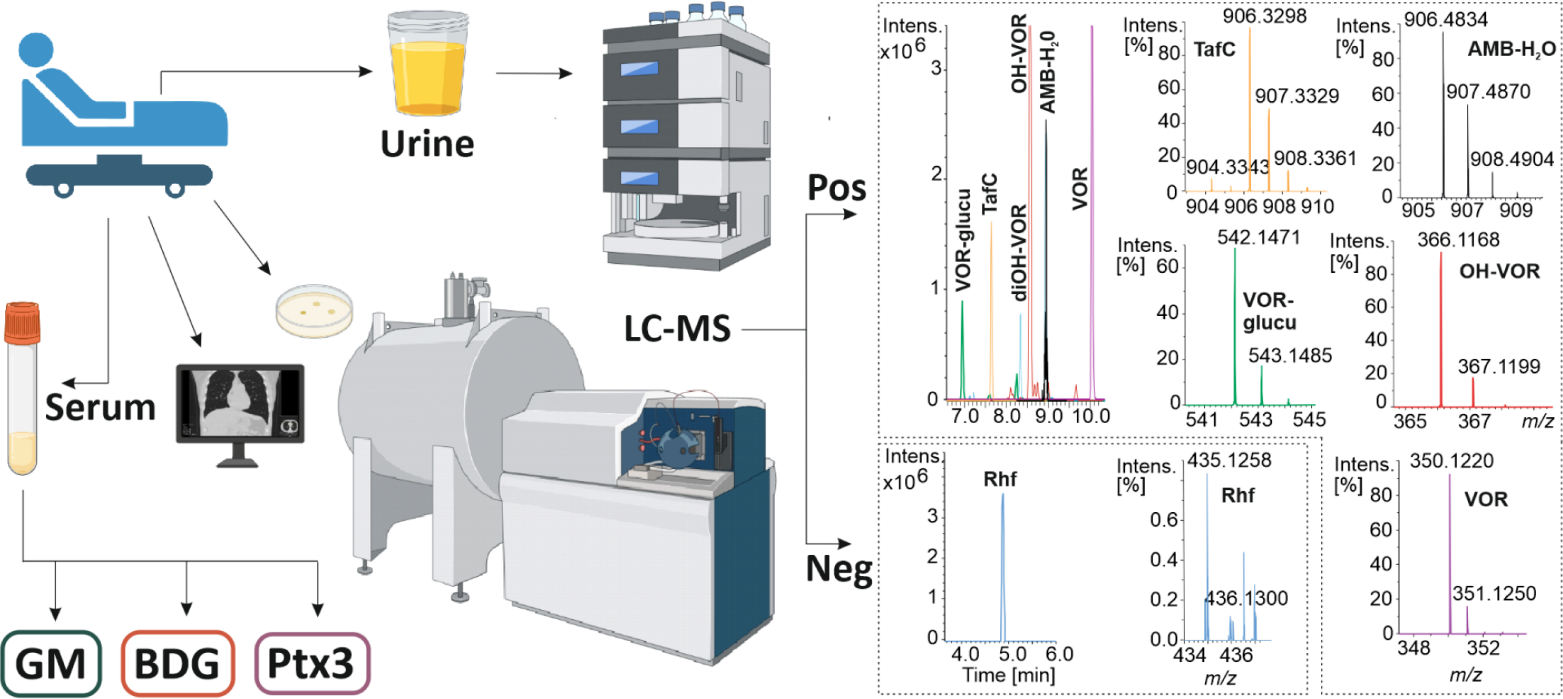
Positive- (Pos) and negative-ion detection (Neg) at day 19 enables parallel analysis of fungal biomarkers and antifungals in the urine. Liquid chromatography and mass spectrometry (LC–MS) analysis of the urine samples goes in line with serum analysis of galactomannan (GM), β-d-glucan (BDG), pentraxin-3 (Ptx3), all via immunoassays, and with X-ray scanning and culture. For illustration, just a selection of metabolites is shown. Rhf, Rhizoferrin; TafC, triacetylfusarinine C; VOR, voriconazole; AMB, amphotericin-B; FoxE, ferrioxamine E; VOR-glucu, voriconazole-glucuronide. This figure was generated in part by Biorender.com.

## 2 Results and Discussion

### 2.1 Course

A 53-year-old woman with angioblastic T-cell lymphoma and neutropenia after allogeneic transplantation and chronic graft-versus-host disease (lower intestinal type) was admitted to the ICU of the University Hospital (Ostrava, Czech Republic) in August 2023. The patient participated in a prospective study comparing the TafC-Rhf-Ptx3 biomarker panel with the performance of the diagnostic standards (sGM and sBDG serology, fungal DNA typing, culture, and radiography).^16^

A high-resolution computed tomography (HRCT) scan taken on day (−1) revealed pulmonary infiltrates, particularly in the right upper lobe (Figure 3A). On day 0 of the 28-day ICU stay, caspofungin (CAS) prophylaxis was initiated at a dose of 70 mg intravenously (iv), followed by 50 mg/day, due to the presence of *Af* DNA (but no *Rm* DNA) in the induced sputum. Levels of sGM and sBDG were elevated at an ODI of 1.45 and a concentration of 626 pg/mL, respectively, indicating ongoing fungal proliferation in the host (Figure 3B). Based on the results of sputum DNA typing, *Aspergillus* was considered the sole causative agent.

**Figure 3:**
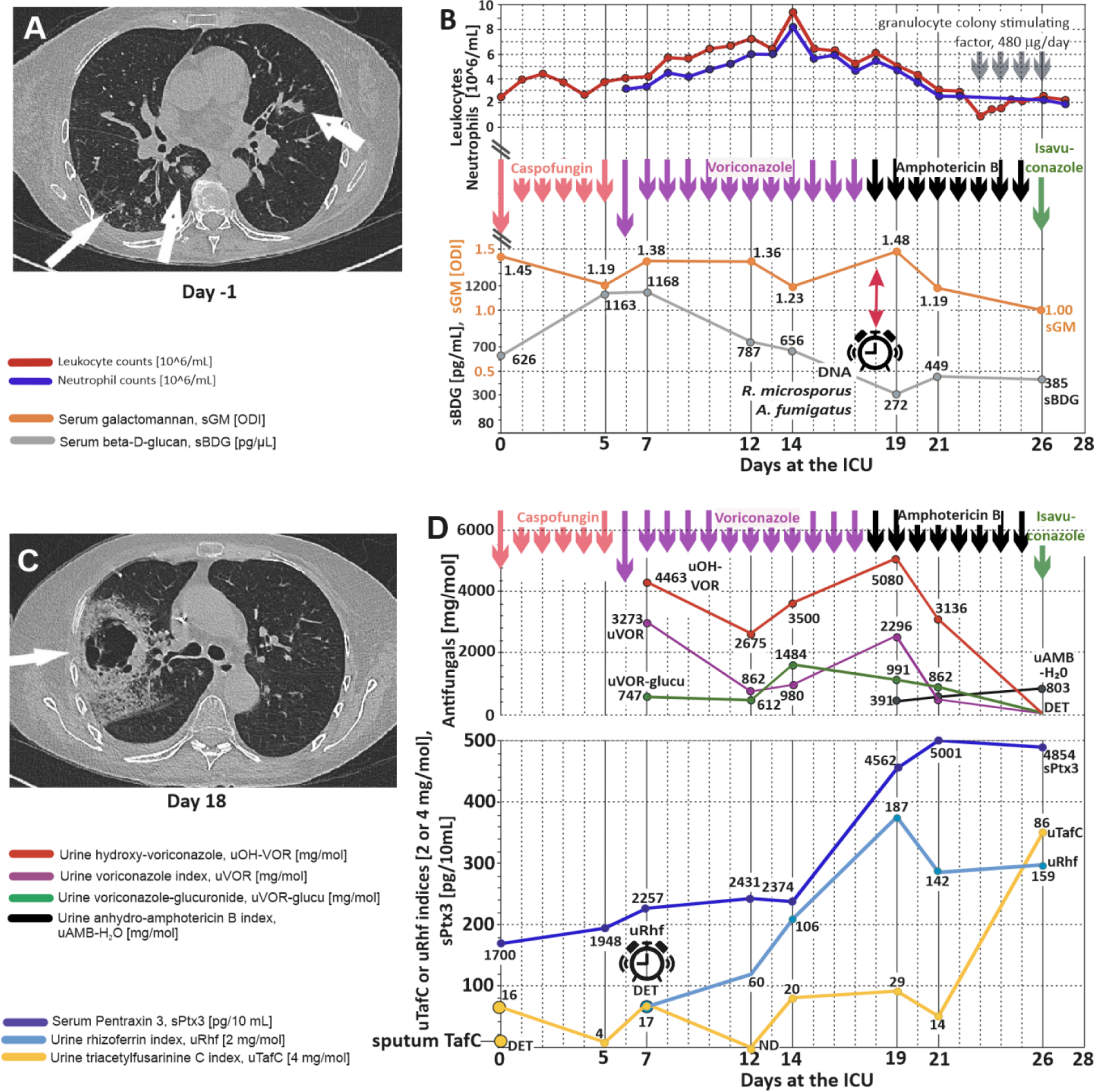
Standard diagnostics with antifungal treatment compared with the next-generation biomarker panel. A: A high-resolution computer tomography (HRCT) scan on the day (−1) indicated a possible invasive mycotic infection, multiple irregular bilateral lung infiltrates (white arrows), and right lung lobe pneumonia. B: Leucocyte, neutrophile, galactomannan (sGM), and 1,3-β-d-glucan (sBDG) serum profiling. The cutoff values for sGM and sBDG positivity were 0.5 ODI and 80 pg/mL, respectively.^18^ Individual doses of caspofungin (70 mg on the first day, then 50 mg/day), voriconazole (VOR, 400 mg/12 h on the first day, then 200 mg/12 h), amphotericin B (AMB 300 mg/day), and isavuconazole (200 mg/8 h for 2 days, then 200 mg/day) are indicated by vertical arrows. The longer arrow indicates the boosting initial dose. C: An HRCT scan of the thorax was taken on day 18, which showed progression of right lobe infiltration, with a 28 mm cavity in segment 1 (S1 in the upper right lobe) and a 6 cm cavity in segments 2–3. D: Serum Ptx3 (sPtx3) was monitored using ELISA (limit of detection, LOD = 22 pg/mL), and urine TafC and Rhf were assessed by mass spectrometry. The LODs defined by mass spectrometry metabolomics were represented by the siderophore method cutoffs (uTafC, 1.1 ng/mL; uRhf, 14.7 ng/mL). Buzzer signs indicate the first molecular detection of *Rm* (DNA or urine Rhf), upon which a diagnosis of mucormycosis can be made. In an HRCT scan, the section is viewed from the bottom with the left side of the Figure is the right side of the patient. Positive ions of antifungal drugs and their metabolites were quantified in the urine (D). VOR (violet) is associated with glucuronide (in green) and hydroxy-VOR (in red). Anhydro-AMB is depicted in black (Figure 2).

Five days of CAS had a negligible effect on serum biomarkers, with only a slight decrease in sGM and an increase in sBDG. The linear CAS metabolite M0 ^17^ was detectable in urine (data not shown). On day 6, the therapy was switched to voriconazole (VOR, 400 mg/12 h/first day, then 200 mg/12 h) and continued for 10 days. Creatinine-adjusted VOR and its metabolites showed stable renal excretion profiles during drug administration (Table 1). During routine therapeutic drug monitoring, the hospital clinical laboratory provided serum VOR concentrations of 2.3 and 5.5 mg/L collected on days 13 and 18, respectively. Throughout the 26 sampling days, serum levels of sGM and sBDG remained consistently elevated, suggesting therapeutic failure. Beginning on day 15, a steady decrease in leukocytes and neutrophils was observed (Figure 3B). The patient’s refractory fungal infection raised the suspicion of an additional occult infection in the lung. As a result, the treatment regimen was changed from VOR to iv liposomal amphotericin B (AMB, 300 mg/day) on day 17, monitored in urine as the AMB anhydro metabolite, and later to iv isavuconazole on day 26 (200 mg/8h for 2 days, then 200 mg/day). On day 18, an induced sputum sample was collected, and *Rm* DNA was detected. A CT scan on day 18 showed a typical nest sign (Figure 3C). The decline in BDG and GM late in the hospital course, despite evident disease progression, is consistent with decline of the *Aspergillus* infection (after some appropriate treatment) and an ascendancy of *Rhizopus* infection; these markers are elevated in an *Aspergillus* infection, but not in infection by Mucorales. The patient was discharged from the hospital on day 28 at her request and expired 7 days after self-discharge.

**Table 1:** Quantitation of Fungal Siderophores and Antifungal Drugs in the Urine^a^

	Concentration [ng/mL]								Crea-normalized concentration [mg/mol]						
Sampling day	TafC	Rhf	VOR	OH-VOR	diOH-VOR	VOR-glucu	Anhydro-AMB	Crea [mmol/L]	TafC	Rhf	VOR	OH-VOR	diOH-VOR	VOR-glucu	Anhydro-AMB
**0**	46	ND	ND	ND	ND	ND	ND	2.9	16	ND	ND	ND	ND	ND	ND
**5**	4	ND	ND	ND	ND	ND	ND	0.9	4	ND	ND	ND	ND	ND	ND
**7**	15	DET	2946	4017	1543	672	ND	0.9	17	DET	3273	4463	1715	747	ND
**12**	ND	84	1207	3744	683	857	ND	1.4	ND	60	862	2675	488	612	ND
**14**	31	159	1470	5250	1091	2225	ND	1.5	20	106	980	3500	727	1484	ND
**19**	32	205	2526	5588	ND	1090	430	1.1	29	187	2296	5080	ND	991	391
**21**	14	142	399	3136	ND	862	442	1.0	14	142	399	3136	ND	862	442
**26**	35	64	DET	DET	ND	2	321	0.4	86	159	DET	DET	ND	4	803

a Absolute concentrations (ng/ml) were creatinine-normalized (mg/mol). Crea, creatinine; TafC, triacetylfusarinine C; Rhf, rhizoferrin; VOR, voriconazole; OH-VOR, hydroxy-voriconazole; diOH-VOR, dihydroxyvoriconazole; VOR-glucu, voriconazole-glucuronide; AMB, amphotericin B; ND, not detected (below limit of detection, LOD); DET, detected (a value between LOD and LOQ). LOQ, limit of quantitation.

### 2.2 Use of Specialized Diagnostics

The experimental Ptx3-siderophore biomarker panel was analyzed in parallel with the routine diagnostics (Materials and Methods section). During the first half of the ICU stay, sPtx3 levels remained constant in a concentration range of 1700 to 2400 pg/mL, which was well below the 2500 pg/mL cutoff that differentiates chronic and invasive stages of a fungal infection.^13^ Between days 14 and 19, the sPtx3 concentration increased from 2374 pg/mL to 4562 pg/mL (Figure 3D) and remained elevated until the end of the stay. The patient’s clinical deterioration (fever, cough, weakness, dyspnea, and chest pain) was consistent with the apparent onset of a massive mixed-type fungal disease.

Sensitive and specific fungal qualitative analysis was provided by urine siderophores (Table 1). TafC was present from the beginning of the ICU stay and was detected in both urine and sputum. We believe that A. fumigatus growth was continuous during most of the 28-day ICU course except for a short window after the initiation of VOR application (Figure 3D). On day 0, the urine TafC/creatinine index was as high as 16. During the ICU stay, the TafC/creatinine index fluctuated between undetectable (Tables 1 and 2) and 86, indicating a partial response to antifungal therapy and continued *Af* proliferation. After the fungistatic CAS, and the initiation of fungicidal VOR and AMB administration, the urine TafC/creatinine index decreased to 4, an undetectable level, and 14 (Table 1). Fast TafC increases between days 5–7 and 12–14 (Figure 3D) indicate an uncontrolled Aspergillus proliferation.

**Table 2:** Limits of Detection and Quantification for Siderophores and Antifungal Drugs^a^

	TafC		Rhf		VOR		AMB	
Validation	Instrumental	Method	Instrumental	Method	Instrumental	Method	Instrumental	Method
LOD [ng/mL]	0.4	1.1	4.9	14.7	0.1	0.4	1.2	3.6
LOQ [ng/mL]	1.1	3.3	16.3	48.9	0.4	1.3	3.7	11.0

a TafC, triacetylfusarinine C; Rhf, rhizoferrin; VOR, voriconazole; AMB, amphotericin B; LOD, limit of detection; LOQ, limit of quantitation

The first positive uRhf sample was obtained on day 7 with an Rhf/creatinine index of 31 (Figure 3D). *Rm* proliferation presumably started between days 5–7, as indicated by the increase in sPtx3 in the subsequent time window. Of note, *Rm* DNA was detected in the sputum 11 days after MS detected the Rhf signal in the urine. It is possible that starting the combination therapy on day 7, when *Rm* was confirmed in the urine, may have improved the patient’s outcome.

In parallel to the fungal biomarkers, creatinine-adjusted antifungals and their metabolites were also monitored in the urine (Figure 2). VOR, hydroxy-VOR, dihydroxy-VOR, VOR-glucuronide, and anhydro-AMB concentrations and creatinine indices are shown in Table 1. Urinary siderophore concentrations correlated with different fungal susceptibilities to CAS, VOR, and AMB. At the beginning of antifungal therapy, expressed by the detection of antifungal drug in the urine, we always observed a decrease in siderophore production. CAS and AMB were not able to completely eliminate siderophore production. Conversely, VOR was the only antifungal potent enough to reduce A. fumigatus growth in a narrow time window before and close to day 12 (Figure 3D). From day 14, both fungi, *Af* and *Rm*, exhibited significant resistance to antifungal therapy. Once AMB was initiated (of the only antifungals used, the only one with activity against mucormycosis), uRhf began to decline.

### 2.3 Strengths and Limitations

The substrate for our assays, urine, is an easily and conveniently obtained abundant specimen for study, and facilitates serial observations, should that prove advantageous. Using microbial siderophores, as demonstrated in this work, invasive fungal infections can be diagnosed earlier than with standard methods. In contrast to the reliability of DNA typing, the *Af* siderophore TafC and *Rm* rhizoferrin reflect active pathogen proliferation at the site of infection. Siderophore secretion correlates with the efficacy of the antifungal drugs used. Siderophores can therefore be used to monitor the relapses of invasive fungal diseases.

This study has several limitations. First, urine-based diagnostics using fungal siderophores leaves specific sites of infection unclear. This limitation can be compensated by parallel sampling close to the site of infection. Second, the iron-rich site of infection may attenuate siderophore production in microbes, potentially causing bias or even false-negative results. Third, the ionization efficiency and stability of the molecular structures of siderophores are largely unknown and require further research.

## 3 Conclusions

This study illustrates the challenges of diagnosing and managing mixed fungal infections in immunocompromised patients, where traditional biomarkers and imaging techniques may not always provide a complete understanding of the patient’s condition. The novel biomarker panel (TafC-Rhf-Ptx3) demonstrated improved accuracy in detecting invasive fungal infections, which is critical for timely and effective management. This is the first clinical report on the use of the Rhf siderophore as a specific biomarker of Mucorales infection. In this study, R. microsporus reached its rapid hyphal growth phase on day 7, which continued until the end of the ICU stay.

We also demonstrated that siderophore secretion correlates with antifungal efficacy and that we can better monitor the fast and striking relapses of the invasive fungal disease in time. We conclude that the early transfer of the LC–MS technology used in this study to matrix-assisted laser desorption-ionization and time-of-flight instrumentation commonly used in clinical laboratories can potentially reduce mortality rates associated with invasive fungal infections and coinfections.

## 4 Materials and Methods

### 4.1 Chemicals

Water (HPLC grade), acetonitrile (ACN), methanol (MeOH), ethyl acetate, and formic acid (FA) were purchased from VWR International (Stříbrná Skalice, Czechia). Standards of ferrioxamine E (FoxE), TafC, and Rhf were purchased from EMC Microcollections (Tübingen, Germany). Deuterated standard voriconazole-*d*_3_ was purchased from Spinchem (Plzen, Czechia). VOR and AMB drug standards were purchased from TCI Europe (Eschborn, Germany) and Scintila (Jihlava, Czechia), respectively.

### 4.2 Study Design

The diagnosis of invasive fungal infection (IFI) was based on the consensus definitions established by the EORTC/MSGERC ICU Working Group. The guidelines of the European Society for Clinical Microbiology and Infectious Diseases and the European Committee for Medical Mycology for pulmonary aspergillosis were used. The IFI was classified as a probable lower respiratory tract mold infection in the absence of a transbronchial biopsy from a primarily sterile site. Sputum samples were collected on days 0 and 18. Urine and serum samples were collected on the same morning. Serum beta-d-glucan (sBDG) measurements were performed using the Fungitell assay (range <0.07–2197 pg/mL, Associates of Cape Cod, Inc., USA). Serum GM (sGM) antigen detection was performed using the *Aspergillus* EIA (Bio-Rad Platelia, France). Optical density index (ODI) sGM > 0.5 and sBDG > 80 pg/mL were the respective cutoff values for a positive test. Serum pentraxin 3 (sPtx3) levels were determined using an ELISA kit from BioVendor (Brno, Czechia). A positive serum sPtx3 assay result, defined by the manufacturer, was 22 pg/mL with linearity over the interval 78–5000 pg/mL.

### 4.3 Urine and Sputum Sample Preparation

Urine and sputum samples from healthy individuals were used to prepare calibration standards for TafC, Rhf, VOR, and AMB at final concentrations of 0.1, 0.5, 1, 2.5, 5, 10, 50, 100, 500, 1000, and 5000 ng/mL (Figure S1). Prior to extraction, all samples (50 μL) were spiked with FoxE and voriconazole-*d*_3_ internal standards and ferric citrate at final concentrations of 50 and 10 ng/mL, and 100 μM, respectively. Control urine, patient urine, and sputum samples were extracted using two-step liquid–liquid extraction.^8^

### 4.4 LC–MS Analysis of Siderophores and Antifungal Drugs

Urine and sputum analyses were performed using a liquid chromatography–mass spectrometry (LC–MS)-based infection metallomics approach described in detail elsewhere.^8,11^ Siderophores were separated on a Dionex UltiMate 3000 high-performance LC system (Thermo Fisher Scientific, MA, USA), and ionized with electrospray ionization (ESI). Reconstituted urine and sputum samples were injected into an Acquity HSS T3/1.8 μm, 1.0 × 150 mm analytical column (Waters, Prague, Czechia). Two different LC methods were used for TafC and Rhf separation (Table S1). Solvent A was water with 0.1% FA, and solvent B was 99% ACN with 0.1% FA.

Siderophores and antifungals were detected using a solariX 12T Fourier-transform ion cyclotron resonance mass spectrometer (Bruker Daltonics, Billerica, MA, USA). MS data were collected in positive and negative ion modes with ESI for TafC and antifungals or Rhf, respectively (Figure 2). The MS parameters were adjusted to optimize the signal intensity and are summarized in Table S1. Besides the parent VOR, three VOR metabolites, i.e., hydroxy-VOR, dihydroxy-VOR, VOR-glucuronide, were detected. AMB and CAS were collected as an anhydro-AMB degradation product or CAS metabolite M0, respectively.^17^ Standard hospital serum VOR therapeutic monitoring was performed on an Acquity BEH C18/1.7 μm, 2.1 × 50 mm analytical column (Waters, Prague, Czechia) in water–ACN gradient (0.1% formic acid buffered with 2 mM ammonium acetate). In multiple reaction monitoring, the VOR eluted at 1.28 min was monitored as *m*/*z* 281.0 fragment generated from a precursor ion *m*/*z* 350.1.

### 4.5 Data Processing and Method Validation

All acquired LC–MS data were processed qualitatively and quantitatively using DataAnalysis v. 5.0 (Bruker Daltonics, Germany) and the CycloBranch software, respectively.^19^ Siderophores and antifungals were quantified against external matrix-matched calibration standards. Using extracted ion chromatograms with a spectral width of 0.005 Da, the responses were summed from the integrated areas of the ferriforms of protonated, sodiated, and potassiated ion species, analyzed in positive ion mode, or the desferriforms of deprotonated and dehydrated ion species, if analyzed in the negative ion mode. The sum of integrated peaks was normalized to the peak area of FoxE, and voriconazole-*d*_3_ for standard siderophores, or AMB and VOR, respectively. Urinary concentrations of siderophores (TafC, and Rhf) were further normalized to the urinary creatinine concentration to obtain siderophore/creatinine index values.^8^ Urine creatinine concentration was determined using an Atellica CH Analyzer (Siemens, Germany) in an Enzymatic Creatinine_3 (ECre3) assay optimized for a working range of 0.18–21.66 mmol/L. Clinical sample preparation methods were validated using control human urine samples according to the US Food and Drug Administration Guidelines for Validation of Bioanalytical Methods (https://www.fda.gov/regulatory-information/search-fda-guidance-documents/bioanalytical-method-validation-guidance-industry) for extraction efficiency (recovery), calibration curve (linearity), limit of detection (LOD), limit of quantitation (LOQ), trueness, precision, and retention time reproducibility (Table S2). Using infection metallomics,^11^ the method limits of detection (LODs) for urine uTafC and uRhf were determined to be 1.1 and 14.7 ng/mL, respectively (Table 2). LC peak shapes detected at the LOD for each analyte are reported in Figure S1. Physico-chemical parameters of analytes separated and detected with liquid chromatography and mass spectrometry are reported in Table S3.

## Supplementary Material



## Data Availability

Raw data can be downloaded from the permanent link https://hdl.handle.net/11104/0365475 and viewed using the open source software CycloBranch, https://ms.biomed.cas.cz/cyclobranch/.

## Notes

Figure S1: Calibration curves of triacetylfusarinine C, rhizoferrin, voriconazole, and amphotericin B and their LC traces at the respective method LODs in urine; Table S1: Mass spectrometry tuning parameters used for data acquisition in positive and negative ion modes; Table S2: LC–MS method validation; Table S3: Physico-chemical parameters of analytes; Table S4: Molar concentrations of fungal siderophores and antifungal drugs quantified in urine samples (PDF)

## Notes

Ethics: The study NCT05860387, entitled “Early diagnosis of invasive pulmonary aspergillosis”, has been registered at ClinicalTrials.gov. It received prior approval from the Ethics Committee for Clinical Trials at the University Hospital of Ostrava (No. 248/22), and informed consent documents were acquired from the participant. Throughout the study, all hospital and research staff adhered to the Good Clinical Practice guidelines of the 2013 Declaration of Helsinki and the general guidelines 86/609/EEC and 200/54/EC 16 for the protection of the European Community due to the handling of potentially infectious materials.
