# Supplementary material for: Detection of Siderophores as a Superior Noninvasive Diagnostic Tool in Unraveling Mixed Fungal Infections
Source: ACS Omega. 2025 May 21;10(21):21908–14. doi: 10.1021/acsomega.5c01914 (PMC12138655; doi:10.1021/acsomega.5c01914)
Supplement: Supplementary file 1 [file ao5c01914_si_001.pdf]

# SUPPORTING INFORMATION

# Detection of siderophores as a superior non-invasive diagnostic tool in unravelling mixed fungal infections

Radim Dobiáš,[1,2#] Milan Navrátil,[3,4#] Rutuja H. Patil,[5] Dominika Luptáková,[5] David A. Stevens,[6] and Vladimír Havlíček[5*]

[1]Department of Bacteriology and Mycology, National Reference Laboratory for Mycological Diagnostics, Public Health Institute in Ostrava, 702 00 Ostrava, Czechia

[2]Institute of Laboratory Medicine, Faculty of Medicine, University of Ostrava, 703 00 Ostrava, Czechia

[3]Department of Haemato-oncology, University Hospital Ostrava, Czech Republic, 703 00 Ostrava, Czechia

[4]Department of Haemato-oncology, Faculty of Medicine, University of Ostrava, 703 00 Ostrava, Czechia

[5]Institute of Microbiology of the Czech Academy of Sciences, 142 00 Prague, Czechia

[6]Division of Infectious Diseases and Geographic Medicine, Stanford University School of Medicine, Stanford, CA 94305, United States

[#]Equal contribution

**[*]Corresponding author:**

Prof. Vladimír Havlíček, Dr.

Institute of Microbiology of the Czech Academy of Sciences

Videnska 1083

142 00 Prague 4, Czechia

Email: vlhavlic@biomed.cas.cz

Phone: +420241062786

# Table of Contents:


| **Figure/Table** | Page |
| --- | --- |
| **Figure S1.** Calibration curves of triacetylfusarinine C, rhizoferrin, voriconazole and amphotericin B and their LC traces at the respective method LODs in urine. | S3 |
| **Table S1.** The solariX FTICR mass spectrometer optimized tuning parameters used for data collection in positive and negative ion modes | S4 |
| **Table S2.** LC-MS method validation. | S4 |
| **Table S3.** Physico-chemical parameters of analytes separated and detected with liquid chromatography and mass spectrometry | S5 |
| **Table S4.** Molar concentrations of fungal siderophores and antifungal drugs quantified in urine samples. | S6 |

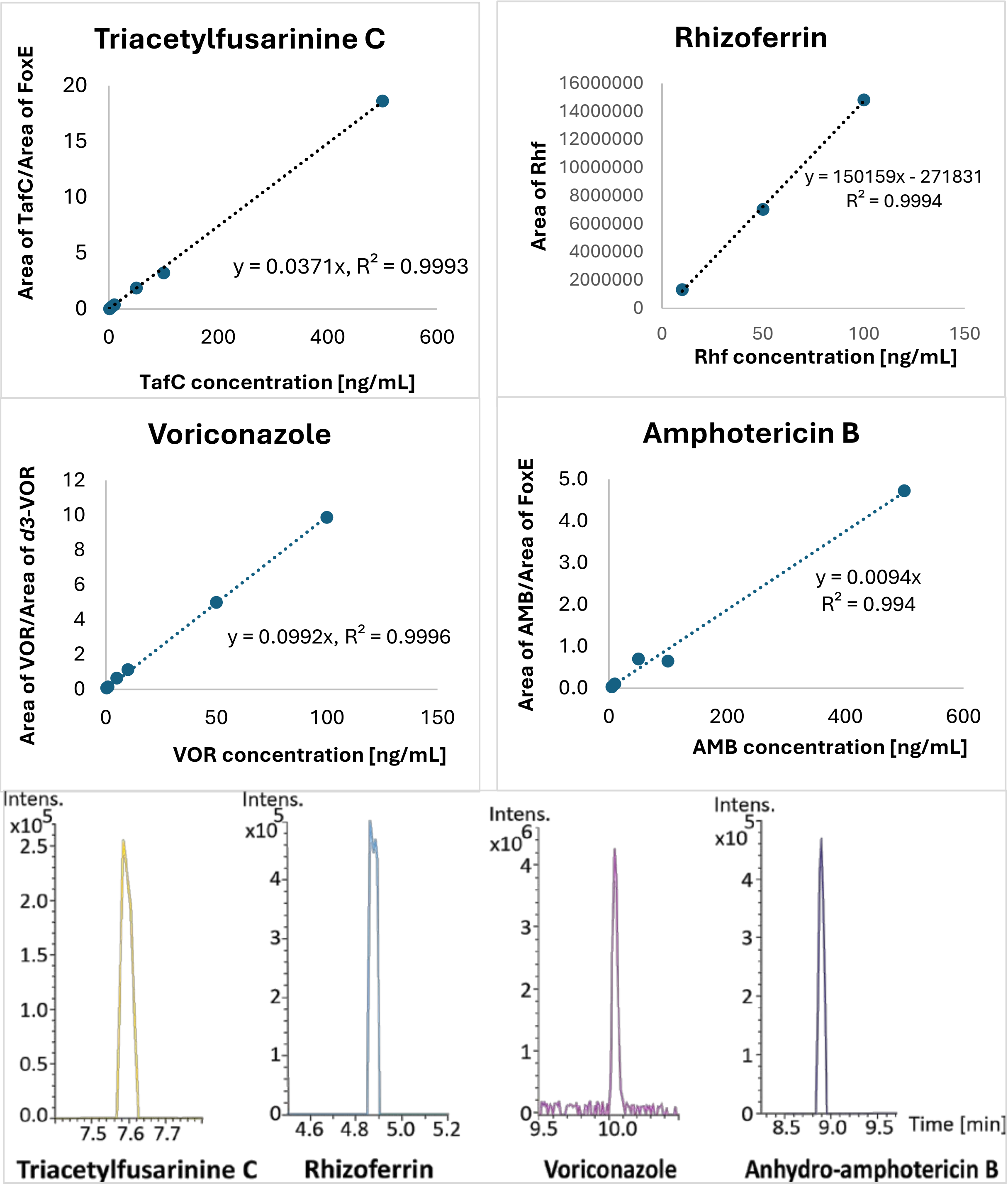


**Figure S1: Calibration curves of triacetylfusarinine C, rhizoferrin, voriconazole and amphotericin B and their LC traces at the respective method LODs in urine (1, 10, 0.5, and 5 ng/mL, respectively).** Control urine, patient urine, and sputum samples were extracted by two-step liquid-liquid extraction [ref. 8 in the main manuscript]. Briefly, the samples were mixed twice by vigorous vortexing with ethyl acetate (150 µL). The organic supernatants were pooled in a separate tube and vacuum dried at 35°C for one hour. Next, ice-cold MeOH (200 µL) was added to the urine and sputum sample residues, vortex-mixed and stored at -80°C for one hour. The samples were centrifuged at 14,000 rpm for 10 minutes at 4°C. The methanolic supernatants were transferred to dried ethyl acetate extracts. The mixed extracts were then vacuum-dried at 35°C for 2 hours. Prior to analysis in duplicate or triplicate (Rhf calibration), the samples were reconstituted in 150 µL of aqueous ACN (15%).

**Table S1. The solariX FTICR mass spectrometer optimized tuning parameters used for data collection in positive and negative ion modes.** VOR, voriconazole; TafC, triacetylfusarinine C; Rhf, rhizoferrin; Pos, positive; Neg, negative;

| Parameter (unit) | VOR | TafC, AMB | Rhf |
|---|---|---|---|
| polarity | Pos | Pos | Neg |
| mass range (low-high m/z) | 200-700 | 500-1200 | 200-1500 |
| ESI capillary voltage (V) | 4800 | 4700 | 4300 |
| end-plate offset (V) | -700 | -700 | -500 |
| nebulizer N2 gas (bar) | 1 | 1.1 | 1.2 |
| drying gas flow rate (L/min) | 3 | 2.5 | 3 |
| capillary exit voltage (V) | 180 | 200 | -220 |
| funnel voltage (V) | 150 | 150 | -150 |
| amplitude (Vpp) | 110 | 160 | 150 |
| skimmer voltage (V) | 15 | 15 | -15 |
| collision cell voltage (V) | -1 | -10 | 4.5 |
| DC bias (V) | 1 | 1 | -1.5 |
| collision radiofrequency (MHz) | 6 | 2 | 2 |
| Q1 selection (m/z) | 400 | 750 | 250 |
| data reduction (%) | 98 | 98 | 97 |
| Flow rate (µL/min) | 50 | 50 | 50 |
| A-B gradient hold 1 | 2% B for 2 min | | |
| A-B slope 1 | 60% B at 8 min | | 70% B at 5 min |
| A-B slope 2 | 99% B at 10 min | | 99% at 8.5 min |
| A-B hold 2 | 3 min at 99% B | | 2.5 min at 99% B |
| A-B linear drop | 0.5 min to 2% B | | |
| A-B hold 3 | 2% B for 1.5 min | | 2% B for 3.5 min |

**Table S2. LC-MS method validation.** TafC, triacetylfusarinine C; VOR, voriconazole; AMB, amphotericin-B; LL, low level concentration (5 ng/mL); HL, high level concentration (50 ng/mL); RSD, relative standard deviation; LOD, limit of detection; LOQ, limit of quantitation; n.d., not determined. LOD, LOQ, linearity, and sensitivity were determined using a set of prepared non-zero calibration standards. Instrumental LOD and LOQ values were defined as the lowest concentrations for which the standard deviation (SD) of the intercept was 3.3 and 10, respectively. The instrumental performance was periodically checked by a system suitability test using an HPLC peptide standard mixture (Sigma-Aldrich, Prague, Czechia).

| Validation parameters | | Analyte | | | |
|---|---|---|---|---|---|
| | | TafC | VOR | AMB | Rhf |
| **Linearity** | | 0.9993 | 0.9998 | 0.991 | 0.9995 |
| **Instrumental LOD (ng/mL)** | | 0.4 | 0.1 | 1.2 | 5.4 |
| **Instrumental LOQ (ng/mL)** | | 1.1 | 0.4 | 3.7 | 16.3 |
| **Trueness, Recovery (%)** | LL | 98 | 113 | 82 | n.d. |
| | HL | 0 | 103 | 115 | n.d. |
| **Precision (RSD %)** | LL | 2 | 4 | 4 | n.d. |
| | HL | 2 | 3 | 2 | n.d. |
| **Reproducibility of retention time (RSD %)** | | 0.05 | 0.03 | 0.07 | 0.1 |

**Table S3. Physico-chemical parameters of analytes separated and detected with liquid chromatography and mass spectrometry.** Rhf, rhizoferrin; TafC, triacetylfusarinine C; VOR, voriconazole; AMB, amphotericin-B; FoxE, ferrioxamine E; CAS, caspofungin; OH-VOR-glucu, hydroxy-O-glucuronide-VOR; VOR-glucu, voriconazole-glucuronide.

| Analyte | Ret. time [min] | Molecular formula | Ion type | Calculated [m/z] | Measured [m/z] | Error [ppm] |
|---|---|---|---|---|---|---|
| Rhf | 4.88 | $C_{16}H_{24}N_2O_{12}$ | $[M-H]^-$ | 435.1257 | 435.1258 | 0.2 |
| TafC | 7.60 | $C_{39}H_{60}N_6O_{15}$ | $[M+Fe-2H]^+$ | 906.3305 | 906.3298 | -0.8 |
| | | | $[M+Fe+Na-3H]^+$ | 928.3124 | 928.3137 | 1.4 |
| VOR | 10.06 | $C_{16}H_{14}N_5OF_3$ | $[M+H]^+$ | 350.1223 | 350.1220 | -0.9 |
| | | | $[M+Na]^+$ | 372.1043 | 372.1039 | -1.1 |
| *d*3-VOR | 10.04 | $C_{16}H_{11}D_3N_5OF_3$ | $[M+H]^+$ | 353.1412 | 353.1410 | -0.6 |
| OH-VOR | 8.52 | $C_{16}H_{14}N_5O_2F_3$ | $[M+H]^+$ | 366.1172 | 366.1168 | -1.1 |
| | | | $[M+Na]^+$ | 388.0992 | 388.0987 | -1.2 |
| diOH-VOR | 8.34 | $C_{16}H_{14}N_5O_3F_3$ | $[M+H]^+$ | 382.1122 | 382.1111 | -2.8 |
| VOR-glucu | 6.89 | $C_{22}H_{22}N_5O_8F_3$ | $[M+H]^+$ | 542.1493 | 542.1471 | -4.0 |
| OH-VOR-glucu | 8.30 | $C_{22}H_{22}N_5O_9F_3$ | $[M+H]^+$ | 558.1442 | 558.1427 | -2.7 |
| Anhydro-AMB | 8.91 | $C_{47}H_{71}NO_{16}$ | $[M+H]^+$ | 906.4846 | 906.4830 | -1.8 |
| FoxE | 6.31 | $C_{27}H_{48}N_6O_9$ | $[M+Fe-2H]^+$ | 654.2671 | 654.2673 | 0.3 |
| Linear CAS | 9.60 | $C_{50}H_{82}N_8O_{16}$ | $[M+H]^+$ | 1051.5922 | 1051.5898 | -2.3 |

**Table S4. Molar concentrations of fungal siderophores and antifungal drugs in urine samples.** TafC, triacetylfusarinine C; Rhf, rhizoferrin; VOR, voriconazole; AMB, amphotericin-B; FoxE, ferrioxamine E; CAS, caspofungin; OH-VOR-glucu, hydroxy-O-glucuronide-VOR; VOR-glucu, voriconazole-glucuronide; not detected (below limit of detection, LOD); DET, detected (a value between LOD and LOQ); LOQ, limit of quantitation.

| Sampling Day | Concentration [nM] | | | | | | |
|---|---|---|---|---|---|---|---|
| | TafC | Rhf | VOR | OH-VOR | diOH-VOR | VOR-glucu | Anhydro-AMB |
| **0** | 51 | ND | ND | ND | ND | ND | ND |
| **5** | 4 | ND | ND | ND | ND | ND | ND |
| **7** | 17 | DET | 8434 | 10996 | 4047 | 1241 | ND |
| **12** | ND | 192 | 3455 | 10249 | 1791 | 1583 | ND |
| **14** | 34 | 364 | 4208 | 14371 | 2861 | 4109 | ND |
| **19** | 35 | 470 | 7231 | 15297 | ND | 2013 | 475 |
| **21** | 15 | 325 | 1142 | 8584 | ND | 1592 | 488 |
| **26** | 38 | 147 | DET | DET | ND | 4 | 354 |
